# Agreement of the performance of equine electrocardiogram recording devices for ECG complexity analysis

**DOI:** 10.1111/evj.70105

**Published:** 2025-11-20

**Authors:** Vadim Alexeenko, Dhruvpal Singh Anchan, Fe Ter Woort, Caroline Ribonnet, Emmanuele van Erck, Celia Marr, Kamalan Jeevaratnam

**Affiliations:** ^1^ University of Surrey Guildford UK; ^2^ Equine Sports Medicine Practice Waterloo Belgium; ^3^ Rossdales LLP Newmarket UK

**Keywords:** annotation, complexity analysis, device agreement, electrocardiogram, horse

## Abstract

**Background:**

Non‐linear equine electrocardiography (ECG) analysis is an actively developing study area which has the potential to lead to novel, artificial intelligence‐based diagnostic tools in equine cardiology. As more ECG recording devices are becoming available, there is a need to ensure results are interchangeable regardless of the equipment used to record the equine ECG.

**Objectives:**

To evaluate the agreement of ECG complexity values obtained using the Televet™ and Equimetre™ systems.

**Study Design:**

Cross‐sectional clinical.

**Methods:**

ECGs were recorded using two devices simultaneously from 37 healthy Thoroughbred horses during routine training. An automated algorithm extracting the ECG segments of acceptable quality extracted 60‐second strips with a stable heart rate in the range 30–100 beats per minute. Threshold‐crossing, beat detection, and feature detection coarse‐graining algorithms were used to annotate the ECG for complexity analysis. Complexity values were corrected to the heart rate using data from 37 horses, and inter‐device agreement was evaluated using Bland–Altman plots and Student's *t*‐test using ECG data from 28 horses that provided sufficient data from both devices.

**Results:**

The results of complexity analysis obtained with beat detection coarse‐graining were independent of the device used at all heart rates. The results obtained with feature detection for heart rates below 75 beats per minute (bpm) and with threshold crossing for heart rates above 75 bpm were significantly different.

**Main Limitations:**

The study relied on convenience sampling, and data analysis was constrained by the availability of ECG data in the heart rate range of interest.

**Conclusions:**

The accurate comparison of ECG complexity analysis results requires consideration of differences between recording devices, heart rates and ECG coarse‐graining techniques.

## 1 INTRODUCTION

Electrocardiography (ECG), heart rate variability (HRV) analysis and advanced non‐linear ECG analysis methods supported by artificial intelligence (AI) techniques are actively researched as promising methods for assessing autonomic nervous system modulation of cardiac activity and diagnostics of cardiovascular diseases. Among these, paroxysmal arrhythmias in exercising horses^1^ are of significant interest due to their potential associations with poor performance and sudden cardiac death.^2^, ^3^ Since differences in signal recording techniques between currently available devices^4^ include not only variation in signal processing but also in electrode placement, ECGs recorded by different devices could be very different in signal quality and morphology. This makes automatisation of ECG interpretation a challenging task, even when using modern AI tools.^5^

An accurate and complete computer interpretation of equine ECGs capturing clinically significant abnormalities might be unnecessary to evaluate the function of the heart. Since pro‐arrhythmic alterations in the cardiac tissue affect the electrical excitation pathways, one might expect that analysis of ECG disorderliness (complexity), or analysis of the ability of the heart to recover after contraction (ECG restitution analysis), might be useful to detect paroxysmal arrhythmias in horses—even without capturing actual arrhythmia episodes.^6^, ^7^, ^8^ Such analyses rely on clinically normal ECG recordings obtained at low heart rates, typically below 100 beats per minute (bpm). To record these ECGs a variety of devices could be used: Televet, Equimetre and others. Televet devices have been successfully used for continuous recording of equine ECG for arrhythmia detection^9^ and for assessment of HRV.^10^ Recordings from both devices were used to conduct ECG disorderliness analyses previously developed for detection of paroxysmal atrial fibrillation.^6^ These analyses estimate the information content in the ECG by converting them into strings of symbols (‘coarse‐graining’ step), and subsequently splitting such strings into sets of sub‐strings using algorithm‐specific rules (‘parsing’ step).^11^, ^12^, ^13^ The size of these sets is directly proportional to the randomness of the strings, thus allowing comparison of disorderliness of original cardiograms.

The objective of this study was to find an agreement on the results of non‐linear analysis of low‐intensity exercise ECGs obtained with two popular equine ECG recording devices: Televet™ (three‐lead system) with a modified base apex configuration and Equimetre™ (single‐lead system), employed simultaneously on healthy Thoroughbred racehorses during routine training sessions. We hypothesise that ECG complexity, once corrected for heart rate, would yield comparable results across different recording devices, despite differences in electrode placement. Rather than matching identical time segments, our approach reflects the practical conditions of veterinary fieldwork, where ECG data are often collected opportunistically. We aim to evaluate whether meaningful and reliable comparisons could be made under these real‐world conditions.

## 2 MATERIALS AND METHODS

### 2.1 Subjects

Thirty‐seven horses were recruited through convenience sampling, with their ECGs obtained as part of routine health assessment. All subjects were Thoroughbred horses of racing age undergoing race training, without any known history of cardiovascular pathology. No additional veterinary evaluation was performed on each individual prior to recruitment. Twelve horses were in training in Belgium with the remaining located in Newmarket, Suffolk. ECG data from 12 of these individuals were also included in previous studies.^9^

### 2.2 ECG recording

**FIGURE 1:**
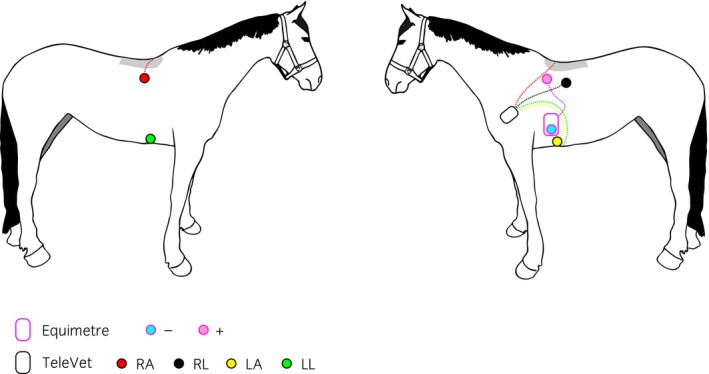
Electrode placement on the horse for Equimetre™ (magenta) and Televet™ (black) devices for the ECG recordings collected in the United Kingdom. ECG, electrocardiography.

The two ECG recording systems were concurrently employed, as shown in Figure 1. These systems encompassed the Televet™ (Engel Engineering GmbH) and the Equimetre™ (Arioneo). The Televet™ Holter system features a recording apparatus equipped with four distinct wire leads interconnected with self‐adhesive gel‐patch ECG electrodes measuring 5 cm in diameter attached to the skin. Electrode placement for the 12 French Thoroughbreds has been reported previously.^9^ Electrode placement for the remaining UK Thoroughbreds was as follows: a modified base‐apex configuration,^4^ with the red electrode (RA) placed on the right side dorsally and the black electrode (RL) on the left side dorsally. In contrast, the yellow electrode (LA) was positioned on the left side just under the girth at the midpoint between the shoulder and elbow and the green electrode (LL) ventrally on the right side. An additional layer of self‐adhesive foam tape was overlaid on top of each self‐adhesive electrode to mitigate the risk of electrode detachment during exercise and reduce artefacts.

Two Equimetre™ products incorporating two substantial conductive electrodes were used interchangeably and both achieve similar electrode placement: a negative ventral electrode and a positive dorsal electrode, connected by a wire (Figure 1). Both electrodes are located on the left side of the horse. The Equimetre™ all‐in‐one girth device comprises a sensor with one electrode positioned at the girth and another which is placed under the saddle on the left side and has been described in detail previously.^10^ The Equimetre™ Saddle and Pad system has two electrodes integrated into a girth and has been described in detail previously.^9^ The electrodes are expansive rectangles, each measuring 15 × 9 cm. The electrodes on the girth features an array of small, soft rubber spikes with conductive properties and an intervening foam layer. These soft spikes penetrate the equine hair coat, enhancing contact surface area and reducing electrode displacement. The second electrode, placed under the saddle, relies on the pressure applied between the saddle and the horse to prevent displacement. Both recording devices have a sampling rate of 500 Hz. To maintain consistency with the previous studies and with the single‐lead configuration of the Equimetre device, recordings, only lead II of Televet device was analysed.

The power analysis indicates that to detect a 5% difference between the two devices (assuming a type I error probability of 5% and a type II error probability of 20%), the cohort includes 16 subjects. As we expected that the drop‐out rate (the probability of the device not recording suitable quality ECG) might be no lower than 50%, 37 subjects were enrolled to ensure sufficient power for the study.

### 2.3 Exercise session

ECG recordings were performed during standard race training sessions, which were approximately 1 hour of exercise. These sessions typically included walking to the exercise areas, then transitioning rapidly to intense exercise. The heart rate exceeded 100 bpm for 15–20 min during intense exercise and was in the 60–100 bpm range for the rest of the time; the transition between these states was rapid and did allow for stable heart rates for the prolonged time. All recordings from all subjects were used to build the dependence of ECG complexity values on heart rate. For between‐device comparisons, we used only the subjects who provided the ECGs from both devices within the investigated heart rate ranges.

### 2.4 ECG segment extraction

ECGs were anonymised and each Televet™ ECG was reviewed subjectively to ensure there were no clinically relevant rhythm disturbances by one of four specialists in equine internal medicine (Fe Ter Worth, Caroline Ribonnet, Emmanuele van Erck or Celia Marr). The original data files were exported to text‐only format (comma‐separated values) files using software supplied by the device manufacturers. The exported files were plotted using a custom R script^14^ and artefact‐free 60‐s strip segments (strips), with stable heart rates between 30 and 120 bpm, were extracted automatically to avoid any subjective selection bias. The heart rate range was selected as previous studies demonstrated the feasibility of detection of alterations associated with paroxysmal atrial fibrillation in normal sinus rhythm ECG recordings at this heart rate range.^6^, ^7^ The extraction was performed by the *FIND_SEGMENT* tool developed by the authors using C++ language independently for Televet™ and Equimetre™ recordings. This tool detects R peaks and analyses R–R intervals as a criterion of ECG segment usability. Strips demonstrating beats with R–R intervals differing by more than 30% deviation from average R–R intervals are excluded, as well as strips with heart rates outside the pre‐defined heart rate range. The 30% threshold was selected empirically as a feasible threshold sufficient to analyse the high‐quality equine ECGs from healthy subjects with low prevalence of premature beats and artefacts. It is sufficient to exclude bradyarrhythmias such as second‐degree atrioventricular block and sinus pauses but will not exclude respiratory sinus arrhythmia. Such an approach was deemed to be appropriate to extract artefact‐free segments with stable heart rates from ECGs that had previously been screened subjectively by experienced equine clinicians. Figure S1 demonstrates the logic and output of the extraction algorithm. Non‐overlapping ECG strips of suitable quality were selected for further analysis. Baseline wander in these strips was eliminated by fitting a polynomial spline at the middle of R–R intervals; the resulting polynomial curve was subtracted from the original ECG. All strips were down‐sampled to a 125 Hz sampling frequency, which was shown previously to be the optimal sampling rate for paroxysmal atrial fibrillation detection using complexity analysis of normal sinus rhythm ECGs.^15^ Since ambulatory ECGs were recorded as a part of normal training activity with only short periods of pre‐exercise and post‐exercise rest, some subjects did not provide artefact‐free ECG strips suitable for analysis from both devices at some heart rate ranges (see Table S1). Therefore, the data from such subjects were used to derive the curves describing complexity value dependence on heart rate but not for between‐device comparisons.

### 2.5 ECG coarse‐graining

**FIGURE 2:**
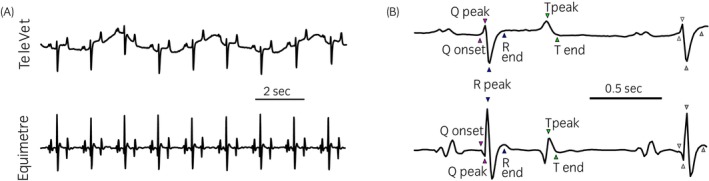
Comparison of ECG recordings by Equimetre™ and Televet™ devices (A) and annotation conventions employed in the study (B). The ‘R end’ feature definition for Equimetre and Televet ECGs is based on the end of voltage increase after the QRS complex. ECG, electrocardiography.

For further analysis, the ECG signal was coarse‐grained (converted to binary text strings) using the method of Feature Detection (FD) described previously.^6^ This method replaces the ECG voltage value by ‘1’ at the onset and peak of the Q wave and at the peak and termination of the R and T waves, with all remaining values replaced by ‘0’. Additional methods of coarse‐graining included Beat Detection (BD), when only the location of the R peak was converted to ‘1’, and the remaining readings were converted to ‘0’, and Threshold Crossing (TC), where values below the threshold (defined as the median voltage value) are converted to ‘0’ and the rest are set to ‘1’. Due to differences in the shape of the ECG signal between devices (Figure 2A), we decided to mark the fiducial points as depicted in Figure 2B.

### 2.6 ECG complexity analysis

Evaluation of ECG complexity after coarse‐graining was performed using a custom implementation of appropriate estimator algorithms implemented in C++. Estimators used in this study include Lempel‐Ziv '76,^13^ abbreviated as LZ76, and Titchener T‐complexity,^16^ abbreviated as Ti. These estimators were selected as it was previously demonstrated their feasibility to detect ECG complexity alterations in normal sinus rhythm ECGs in humans and horses with a history of paroxysmal atrial fibrillation.^6^, ^17^ The basic principle of either evaluator involves splitting a symbolic string into a set of substrings needed to build the original string by some computer capable of copying and extension operations. The higher number of substrings identified by the algorithm indicates higher complexity of the analysed string and therefore higher disorderliness of it. The detailed discussion of both algorithms may be found in publications referenced above. To eliminate the dependency of complexity values on the length of the source string (*n*), LZ76 complexity values were normalised to the *n*/log_2_(*n*) value,^11^ and for Ti complexity average entropy values were used.

### 2.7 Correction of complexity values to the heart rate

**FIGURE 3:**
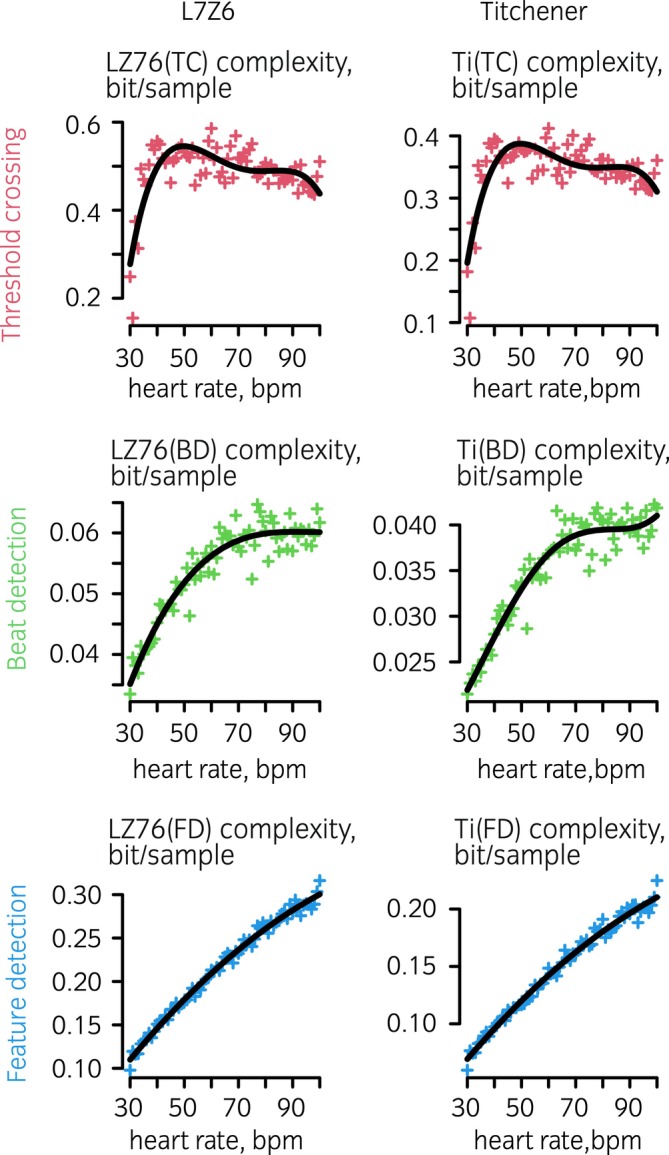
Dependence of complexity values on the heart rates for different techniques (irrespective of device used). All data grouped by heart rate, with 1 bpm steps. Curves are optimal polynomial fits, fourth order for TC, third and fourth order for LZ76(BD) and Ti(BD) and second‐order FD data. BD, beat detection; FD, feature detection; TC, threshold crossing.

In the previous studies, it was observed that ECG complexity values are non‐linearly influenced by the heart rate.^18^ To reduce the influence of heart rate on the complexity values, we fitted the optimal polynomial curves using *polyfit* and *optimize* R functions in *pracma* and *stat* packages (Figure 3). The optimally fitted polynomials (fourth order for TC, third and fourth order for LZ76(BD) and Ti(BD), and second order for FD) to the averaged complexity data values (Figure 3) were selected using Akaike's ‘An Information Criterion’ tool in R, and the resulting curve was subtracted from individual data sets. This operation transformed data to normally distributed residuals, which did not depend on the heart rate but only on the device used. Only the data from horses that provided ECG strips for both devices were used for inter‐device comparisons.

### 2.8 Data analyses

Parametric data are expressed as mean ± standard deviation of mean. Statistical analyses and plotting were done using GNU R 4.1.2.^14^ For comparison of the two devices, data from 28 horses in which ECG data of adequate quality were available from both devices were used. The median number of strips per horse was 18 for Televet™ and 14 for the Equimetre™ device, and average heart‐rate complexity values for each horse were used for inter‐device comparison. The assumption that the mean differences were normally distributed was checked by plotting histograms, and Bland–Altman plots (the difference between measures versus the average of the measures from the two devices for each individual) were used to assess whether the variation was dependent on the magnitude of the measurements. A two‐sided *t*‐test (using Welch's correction for unequal variances) was used for two‐group comparisons with significance between data sets accepted at *p* < 0.05.

## 3 RESULTS

**FIGURE 4:**
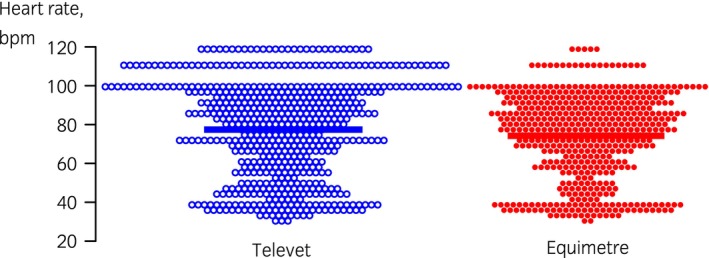
Distribution of heart rates in automatically extracted ECG strips. Each circle represents a single strip; horizontal bars show average heart rates. ECG, electrocardiography.

The total number of automatically extracted ECG strips exceeded 500 for each device. Since at heart rates exceeding 100 bpm, data availability was limited (Figure 4), the further analysis was limited to recordings with heart rates in the 30–100 bpm range. The breakdown of the number of strips and clinical and signal quality evaluation is shown in Table S1.

### 3.1 Mean complexity values are dependent on the heart rate and device used

**FIGURE 5:**
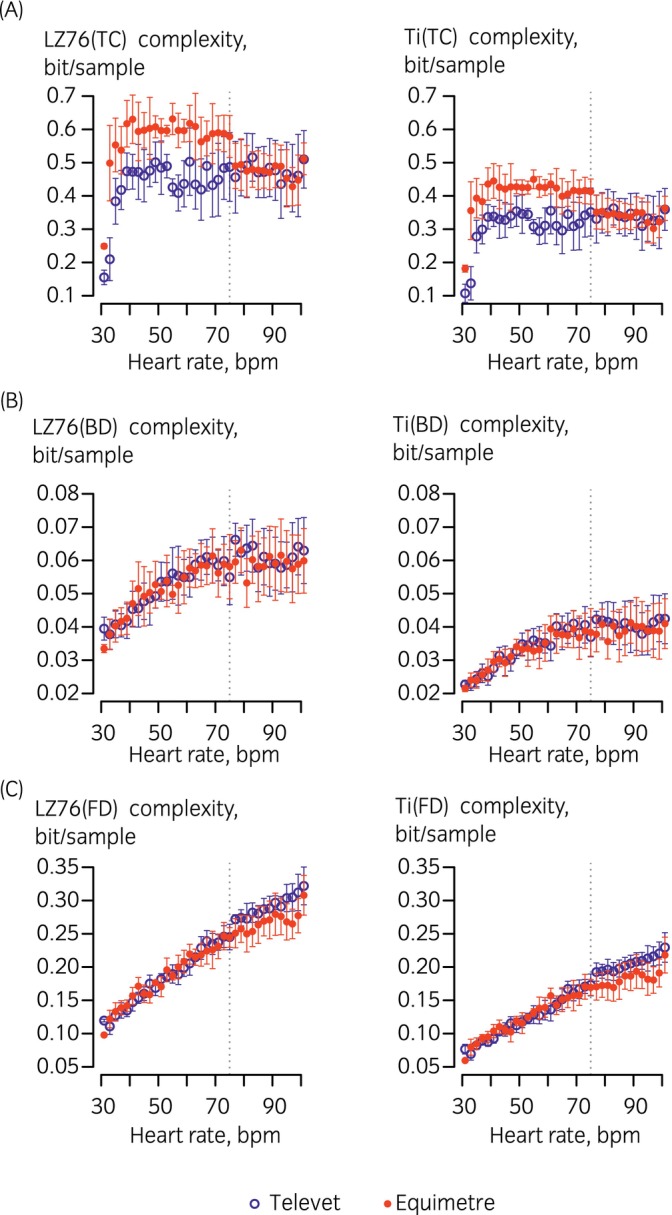
Dependence of LZ76 and Titchener ECG complexity on the heart rate for Equimetre™ and Televet™ devices. (A) Threshold crossing coarse‐graining. (B) Beat detection coarse‐graining. (C) Feature detection coarse‐graining Vertical dotted line at 75 bpm indicated the heart rate at which the behaviour of complexity estimators changes. Equimetre™ data are shown in red, Televet™ data are shown in blue. ECG, electrocardiography.

To estimate the dependency of complexity values on the device type at different heart rates, we plotted average complexity values for given heart rates for both devices (Figure 5). We observed that while the dependency of complexity values on the device was very similar for both devices, in certain combinations of coarse‐graining techniques, complexity estimators, and heart rate, the difference was easily observable. While beat detection (Figure 5B) and feature detection (Figure 5C) coarse‐graining‐processed data were very similar across the 30–75 bpm heart rate range, the complexity of ECGs processed with threshold‐crossing coarse‐graining demonstrated the opposite tendency: marked difference at low heart rates and similarity at heart rates exceeding 75 bpm (Figure 5A).

### 3.2 Agreement of average ECG complexity estimates using Equimetre™ and Televet™ devices

**FIGURE 6:**
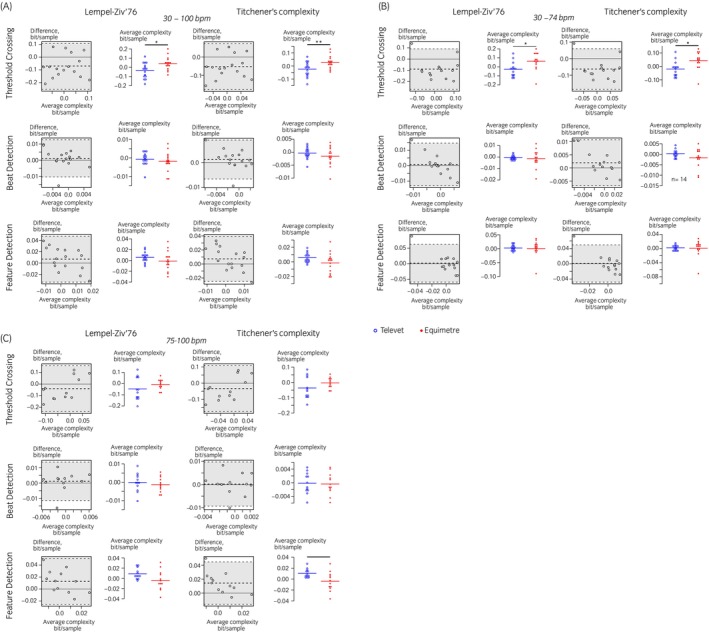
Bland–Altman plots for agreement between devices using different coarse‐graining techniques and different complexity estimators at different heart rate ranges. (A) Full heart rate range (30–100 bpm), *n* = 16. (B) Low heart rate range (30–75 bpm), *n* = 14. (C) High heart rate range (75–100 bpm), *n* = 12. Equimetre™ data are shown in red, Televet™ data are shown in blue.

To evaluate the agreement between devices, we conducted the analysis using Bland–Altman plots and *t*‐tests, using several heart rate ranges: 30–100 , 30–74, and 75–100. The cut‐off of 75 bpm reflects the stepwise discontinuity in complexity values observed for TC complexity (Figure 5A). The results of analyses (Figure 6 and Table 1) demonstrated that there are no statistically significant differences for any combination of heart rate range and complexity estimator used if the BD coarse‐graining technique was employed. However, there was a statistically significant difference between devices for TC coarse‐graining at heart rate ranges 30–100 and 30–74 bpm (Figure 6A,B). The situation was reversed for FD coarse‐graining: there was no difference at the heart rates 30–100 bpm and for 30–75 bpm, while at the heart rate range 75–100 bpm there was a statistically significant disagreement between devices using the Ti complexity estimator (Figure 6C).

**TABLE 1:** Agreement between average heart‐rate corrected complexity values obtained for different heart rates, devices, and metrics (complexity estimator and coarse graining technique combination).

Metric	Device	Complexity at 30–100 bpm, bit/sample (*n* = 16)	Complexity at 30–75 bpm, bit/sample (*n* = 14)	Complexity at 75–100 bpm, bit/sample (*n* = 12)
LZ76(TC)	Televet™	−0.0332 ± 0.0798	−0.0268 ± 0.0804	−0.0474 ± 0.102
	Equimetre™	0.0394 ± 0.0711	0.0622 ± 0.0945	−0.00786 ± 0.0468
		*p* = 0.01	*p* = 0.01	*p* = 0.2
LZ76(BD)	Televet™	−0.000738 ± 0.00347	−0.000564 ± 0.00209	−0.000133 ± 0.00507
	Equimetre™	−0.00184 ± 0.00499	−0.00138 ± 0.00756	−0.00129 ± 0.00423
		*p* = 0.5	*p* = 0.7	*p* = 0.6
LZ76(FD)	Televet™	0.00575 ± 0.00896	0.00246 ± 0.00974	0.00869 ± 0.0104
	Equimetre™	−0.00116 ± 0.0176	0.000406 ± 0.0302	−0.00414 ± 0.0196
		*p* = 0.2	*p* = 0.8	*p* = 0.06
Ti(TC)	Televet™	−0.0246 ± 0.0566	−0.0196 ± 0.0556	−0.0361 ± 0.0730
	Equimetre™	0.0276 ± 0.0477	0.0432 ± 0.0656	−0.00299 ± 0.0347
		*p* = 0.008	*p* = 0.01	*p* = 0.2
Ti(BD)	Televet™	−0.000503 ± 0.00218	0.000302 ± 0.00181	−0.000465 ± 0.00311
	Equimetre™	−0.00167 ± 0.00335	−0.00170 ± 0.00457	−0.000706 ± 0.00297
		*p* = 0.3	*p* = 0.1	*p* = 0.8
Ti(FD)	Televet™	0.00581 ± 0.00637	0.00190 ± 0.00693	0.0104 ± 0.00827
	Equimetre™	−0.00148 ± 0.0139	0.00103 ± 0.0239	−0.00395 ± 0.0175
		*p* = 0.07	*p* = 0.9	*p* = 0.02

*Note*: Data in the table are shown are as the mean ± standard deviation.

Abbreviations: BD, beat detection; FD, feature detection; TC, threshold crossing.

## 4 DISCUSSION

Increasing computing power of consumer electronics allows a variety of devices to be used for equine ECG recordings: this includes fitness trackers and smartphones.^18^, ^19^, ^20^, ^21^ Such devices, while not intended to be used as clinical cardiac diagnostic tools, might still provide data usable for diagnostic purposes. The current clinical practice relies on detection of abnormalities in long (hours or days) electrocardiograms, which makes the quality of recording an important issue, as it affects the ability of human or AI interpreters to classify heartbeats as normal or pathological. On the contrary, non‐linear ECG analysis employed in this study neither requires long ECG recordings nor is aimed at detection of abnormalities. On the contrary, it aims at capturing alterations in signal conduction through cardiac tissue at low heart rates, and in relatively short episodes of ECG: 60 s or less. This has an important consequence: such a signal might be obtained using a device capable of short ECG recordings, or by splitting longer telemetric ECGs into usable fragments, as in the present study, with a rather simple rule‐based algorithm applied for signal quality assurance.

This study clearly demonstrates the influence of ECG pre‐processing (coarse‐graining) technique on the result of complexity analysis. Unlike analysis of complexity of neuronal spike trains^22^ or of EEG,^23^ the analysis of ECG disorderliness requires precise capturing of certain fiducial points, which include not only easy identifiable peaks of the waveforms, but more challenging location of waveform onset and termination points. This problem is most acute for low amplitude waveforms (e.g., P wave, Figure 2B) due to very gradual decrease of voltage signal at these points and in recordings which are noisy or have issues with unstable baseline. Therefore, a simple threshold‐crossing coarse‐graining algorithm perfectly suitable for EEG or spike train analysis is inadequate for ECGs: if threshold is set close to the isoelectric line it would not capture the precise location of any peak, and at the same time it would be very susceptible to any baseline wander or high‐frequency noises which affect that baseline. A perfect example of influence of baseline noise and wander is demonstratable by differences of complexity values provided by LZ 76 and Ti estimators for Televet™ device and Equimetre™ devices at heart rates below 75 bpm (Figure 5A).

This necessitates the use of techniques which would be insensitive to baseline wander and noise. The extreme variation of such technique, BD, has demonstrated compatible results at all heart rates and all complexity estimators (Figure 5B and Table 1). The previously proposed FD coarse‐graining technique which captures locations of several fiducial points on the waveform was expected to be likewise insensitive to the device used. Contrary to this assumption, the agreement of complexity estimates between devices at 75 bpm markedly changed, not only when using the TC coarse‐graining method, but also for FD coarse graining (Figure 5A,C and Table 1). The results of FD‐based complexity estimations at heart rates >75 bpm were consistently and statistically significantly different between devices when using Ti complexity estimator. These effects might be conveniently explained by changes in ECG complexity values for the Equimetre device, which are stepwise for TC coarse‐graining (Figure 5A) but gradual for FD coarse‐graining (Figure 5C). It might be speculated that such changes might reflect some physiological phenomena, but a more plausible explanation would be that they are due to non‐linear nature of ECG complexity estimation algorithms: influence of noise on TC and FD complexity values might be different above and below 75 bpm. Such behaviour of these algorithms has to be accounted for in subsequent studies. It might be expected that sampling rate, signal filtering and coarse‐graining technique might affect the existence of such change and its magnitude.

The major limitation of this study is the use of ambulatory ECGs obtained during standard race training procedures. This restricted the availability of low heart rate ECGs for device performance comparison, with only 40% of recorded data files allowing extraction of suitable segments. Whilst this acceptance rate is low, it was deemed acceptable as the study's aim was to compare agreement between two devices rather than develop a diagnostic technique. Future studies aimed at deep understanding of equine ECG complexity dependence on heart rate and on inter‐device differences might benefit from pre‐planned ECG samples collected specifically for the purpose of thorough coverage of different heart rates when the analysed data from different devices would overlap.

Although many horses now have ECGs routinely recorded during exercise, resting ECGs offer several advantages for clinical applications. Resting ECGs are easier to perform, less technically challenging, and subject to less artefact compared to exercise ECGs. These factors make resting ECGs particularly valuable for developing standardised analytical approaches. For the purpose of medical diagnosis, our goal is to develop techniques derived from resting ECGs to predict rhythms such as paroxysmal atrial fibrillation. The typical resting heart rate of a horse is close to 30 bpm, well below 75 bpm. This lower heart rate range is crucial for our analysis, as it allows for the detection of subtle alterations in signal conduction without the confounding influence of higher heart rates. While this approach may not be essential for all types of arrhythmias, it could prove particularly beneficial for diagnosing more critical conditions such as paroxysmal ventricular arrhythmias. The major insight we derive from this study is that complexity analysis using the FD coarse‐graining scheme, which combines low sensitivity to baseline wander and noise with capturing important events of the cardiac cycle did provide the same results for both devices at heart rates 30–75 bpm, which is of particular interest for the detection of paroxysmal atrial fibrillation.

This study for the first time has evaluated the dependence of FD complexity values across a range of heart rates. Previously,^6^ it was assumed that this dependence is linear; however, our new observations suggest it is not so. This observation might be accounted for in calculations of heart rate corrected complexity values obtained using FD coarse graining. This might be essential as it might affect the diagnostic outcome of arrhythmia detection. However, the degree of non‐linearity is low, especially at heart rates below 75 bpm, and further detailed study will be required to derive an appropriate correction formula.

## 5 CONCLUSION

The study clearly demonstrated that when using the BD coarse‐graining technique, both Equimetre™ and Televet™ devices could be used interchangeably for the purpose of ECG complexity analysis. At the same time, complexity analysis using TC and FD coarse‐graining requires careful consideration of the device type and heart rate to produce compatible estimates. It could be expected that this technique could be successfully applied for the analysis of the ECGs collected by other devices provided they use similar electrode placement and data filtering settings to either the Equimetre™ or Televet™ device.

## FUNDING INFORMATION

This study was supported by the Horserace Betting Levy Board grant vet/prj/800 (2021).

## CONFLICT OF INTEREST STATEMENT

The authors declare no conflicts of interest.

## AUTHOR CONTRIBUTIONS

**Vadim Alexeenko:** Conceptualization; methodology; software; data curation; investigation; validation; formal analysis; visualization; writing – review and editing; writing – original draft. **Dhruvpal Singh Anchan:** Methodology; data curation; visualization; writing – review and editing; writing – original draft. **Fe Ter Woort:** Data curation; investigation; writing – review and editing. **Caroline Ribonnet:** Writing – review and editing; data curation; methodology. **Emmanuele van Erck:** Writing – review and editing; data curation; methodology. **Celia Marr:** Conceptualization; methodology; writing – review and editing; funding acquisition; supervision. **Kamalan Jeevaratnam:** Project administration; resources; writing – review and editing; methodology; conceptualization; funding acquisition; supervision.

## DATA INTEGRITY STATEMENT

Vadim Alexeenko and Kamalan Jeevaratnam had full access to all the data in the study and take responsibility for the integrity of the data and the accuracy of the data analysis.

## ETHICAL ANIMAL RESEARCH

Ethical approval for this study was granted by the NASPA Sub‐Committee, School of Veterinary Medicine, University of Surrey (NASPA 2021‐08).

## INFORMED CONSENT

Explicit owner consent for inclusion of animals in this study was not obtained. Owners or their agents were made aware that case information may be used for research in general.

## Supporting information


**Figure S1:** Automated extraction of ECG strips for further analysis from ambulatory telemetric recordings.

**Table S1:** Clinical ECG interpretation and signal quality notes.

## Data Availability

The data that support the findings of this study are available upon reasonable request from the corresponding author. Open data sharing exemption granted by the editor.
