# Supplementary material for: Agreement of the performance of equine electrocardiogram recording devices for ECG complexity analysis
Source: Equine Vet J. 2025 Nov 20;58(5):1336–44. doi: 10.1111/evj.70105 (PMC13447865; doi:10.1111/evj.70105)
Supplement: Supplementary file 1 [file EVJ-58-1336-s002.pdf]

Table S1: Clinical ECG interpretation and signal quality notes.

#	Horse ID	Subjective ECG interpretation	Televet™ quality notes	Televet™ strips extracted	Equimetre™ quality notes	Equimetre™ strips extracted
1	1016	3 wPC during recovery post exercise	Good; Unstable baseline	12	Poor, frequent artefact	0
2	1018	1 wPC in warm-up	Good	7	Acceptable; moderate artefact	3
3	1019	No Abnormalities Detected	Good	28	Acceptable; moderate artefact	20
4	1020	No Abnormalities Detected	Good	34	Good	21
5	1021	No Abnormalities Detected	Good	3	Good	3
6	1022	3 nPC P& 2 wPC in recovery post exercise	Good	12	Poor, frequent artefact	0
7	1030	2 wPC gallop during exercise	Good	28	Electrode disconnection	0
8	1032	No Abnormalities Detected	Acceptable	17	Poor, frequent artefact	0
9	1033	No Abnormalities Detected	Acceptable	8	Poor, frequent artefact	2
10	1034	No abnormalities detected, poor quality at gallop during exercise	Good; Unstable baseline	15	Poor, frequent artefact	1
11	1039	No Abnormalities Detected	Good; Unstable baseline	17	Electrode disconnection	0
12	1040	No Abnormalities Detected	Good; Unstable baseline	14	Acceptable; moderate artefact	7
13	1042	No Abnormalities Detected	Good	37	Poor, frequent artefact	4
14	1043	No Abnormalities Detected	Good	21	Good; infrequent artefact	14
15	1044	1 nPC in warm-up, 3 wPC in gallop and in post exercise recovery	Good	13	Poor, frequent artefact	0

16	1045	1 nPC & 1 wPC in gallop; 3 wPC in recovery post exercise	Good; infrequent artefact	14	Good; infrequent artefact	13
17	1047	No Abnormalities Detected	Good	14	Good	11
18	1048	1 nPC & 1 wPC in gallop; 2 wPC in recovery post exercise	Good	20	Poor, frequent artefact; electrode disconnection	0
19	1049	2 wPCwarm-up, 2 wPC in gallop, 1nPC, 1 wPC & run of wPC in recovery post exercise	Good; Unstable baseline	27	Good	20
20	1050	1 wPC in gallop, 1 wPC in recovery post exercise	Good	22	Poor, frequent artefact	2
21	1052	No Abnormalities Detected	Good	30	Poor, frequent artefact	5
22	1053	8 nPC in warm-up, 4nPC in gallop & 2 nPC in recovery post exercise	Good; Unstable baseline	23	Poor, frequent artefact	0
23	1055	No Abnormalities Detected	Good; Unstable baseline	8	Poor, frequent artefact	0
24	1056	No Abnormalities Detected	Good; Unstable baseline	13	Good	5
25	1058	No Abnormalities Detected	Good	19	Acceptable; moderate artefact	11
26	1059	2 nPC in recovery post exercise	Good	21	Good	30
27	1060	No Abnormalities Detected	Good	39	Good	44
28	1061	No Abnormalities Detected	Good	31	Good	42
29	1062	4 wPC in gallop and in recovery post exercise	Good	35	Good	31
30	1063	No Abnormalities Detected	Good; Unstable baseline	6	Good	20
31	1064	No Abnormalities Detected	Good	1	Good	16
32	1065	No Abnormalities Detected	Good; Unstable baseline	22	Good	16

33	1066	No Abnormalities Detected	Good	24	Good	26
34	1067	No Abnormalities Detected	Poor, electrode disconnection	3	Good	25
35	1068	2 nPC in gallop, poor quality at gallop	Poor, electrode disconnection	7	Good	31
36	1069	No Abnormalities Detected	Poor, electrode disconnection	5	Good	14
37	1070	No Abnormalities Detected	Good	17	Good	13

Abbreviations: wPC = wide premature complex, nPC = narrow premature complex; quality notes: good – low artefact rate (disconnection, shivering, etc) rate; acceptable – some artefacts present, poor – very frequent artefacts
